# Genetic Diversity and Molecular Epidemiology of *Mycobacterium tuberculosis* Complex Clinical Isolates in New Brunswick, Canada—A Retrospective Chart Review

**DOI:** 10.3390/pathogens15010115

**Published:** 2026-01-20

**Authors:** Isdore Chola Shamputa, Derek J. Gaudet, Jason McKinney, Kim Barker, Hafid Soualhine, Catherine Yoshida, Meenu Kaushal Sharma, Duncan Webster

**Affiliations:** 1 Faculty of Nursing and Health Sciences, University of New Brunswick, Saint John, NB E2E 4L5, Canada; 2 Department of Psychology, University of New Brunswick, Saint John, NB E2E 4L5, Canada; derek.gaudet@unb.ca; 3 Department of Laboratory Medicine, Division of Medical Microbiology, Saint John Regional Hospital, Saint John, NB E2L 4L2, Canada; jason.mckinney@horizonnb.ca (J.M.); duncan.webster@horizonNB.ca (D.W.); 4 Department of Health, Government of New Brunswick, Saint John, NB E1A E9H, Canada; kimberley.barker@gnb.ca; 5 National Reference Centre for Mycobacteriology, National Microbiology Laboratory, Public Health Agency of Canada, Winnipeg, MB R3E 3R2, Canada; hafid.soualhine@phac-aspc.gc.ca (H.S.); meenu.sharma@phac-aspc.gc.ca (M.K.S.); 6 Department of Medical Microbiology and Infectious Diseases, University of Manitoba, Winnipeg, MB R3E 0J9, Canada; 7 Applied Genomics Innovation for Laboratory Excellence Section, National Microbiology Laboratory, Public Health Agency of Canada, Winnipeg, MB R3E 4R2, Canada; catherine.yoshida@phac-aspc.gc.ca; 8 Dalhousie Medicine New Brunswick, Dalhousie University, Saint John, NB E2L 4L5, Canada; 9 Department of Medicine, Division of Infectious Diseases, Saint John Regional Hospital, Saint John, NB E2L 4L2, Canada

**Keywords:** *Mycobacterium tuberculosis* complex, clinical isolates, tuberculosis, epidemiology, genetic diversity, New Brunswick

## Abstract

The incidence of tuberculosis disease (TBD) in New Brunswick (NB) is low but has been rising over the past decade. Analyzing these trends can help identify specific risk factors and transmission patterns to guide targeted public health strategies. This study aimed to provide a comprehensive and detailed characterization of TBD in NB by examining data from 1 January 2002, to 31 December 2024. All TB patients with *Mycobacterium tuberculosis* complex (MTBC) clinical isolates identified in NB healthcare facilities were eligible for inclusion in the study. We analyzed demographic, drug susceptibility, and 24-locus Mycobacterial Interspersed Repetitive Unit-Variable Number Tandem Repeat (MIRU-VNTR) data from 166 patients. Most MTBC isolates were pan-susceptible to first-line anti-tuberculosis drugs (90.9–98.1%), with 2.4% showing multidrug resistance. The MIRU-VNTR demonstrated a high discriminatory power of 0.9982 and a low clustering rate of 20.4%. Two samples from the same patient, collected seven years apart, showed different genetic profiles, suggesting that the second episode was a new infection. The most prevalent MTBC lineage was East African Indian (*n* = 23, 13%). This study provides early insights into TB trends in NB, including what may be the first recorded case of TB reinfection in NB. Our findings will help guide future TB research, policies, and public health interventions in the region.

## 1. Introduction

Tuberculosis (TB), caused by *Mycobacterium tuberculosis*, a member of the *M. tuberculosis* complex (MTBC), continues to pose a serious threat to global public health. In 2024, an estimated 10.7 million people suffered from TB, with 1.23 million losing their lives to the disease [1]. While most TB cases occur in low- and middle-income countries, prevention and control efforts remain essential in high-income countries like Canada due to the interconnected nature of global health. Although TB rates in Canada remain low compared to global figures, the incidence showed a gradual increase between 2015 and 2023, rising slightly from 4.6 to 5.5 cases per 100,000 population [2]. A similar upward trend was observed in New Brunswick (NB), one of the four Atlantic provinces of Canada, where the incidence of TB climbed from 0.4 to 2 cases per 100,000 between 2013 and 2022 [3]. The province of NB borders Quebec to the north, Nova Scotia to the east, and the U.S. state of Maine to the south and west. The 2021 Canadian census reported that NB had a population of 775,610, accounting for about 2% of Canada’s total population [4]. Immigration is a key driver of population growth in NB, with the proportion of immigrants increasing from 3.1% in 2001 to 5.8% in 2021 [4].

The proportion of reported TB cases among foreign-born individuals in Canada increased from 65% in 2001 to 72% in 2020 [5], rose to 79% in 2023 [2], and reached 82.9% in 2024 [6]. A similar trend was observed in NB, where the proportion increased from 33% in 2013 to 100% in both 2022 and 2023 [7]. In 2022, Canada reaffirmed its 2014 commitment to eliminating TB as a public health threat by signing the World Health Organization (WHO) Action Framework for Low-Incidence Countries, developed under the Stop TB Partnership. The framework sets a pre-elimination target of fewer than 1 case per 100,000 people by 2035 and a full elimination goal of reducing the TB incidence to fewer than 1 case per million population by 2050 [8].

Understanding the unique epidemiology of TB in different regions of the country is important for designing and implementing targeted interventions and prevention strategies. These efforts can help curb the development and spread of TB disease (TBD) and drug resistance, lower the overall disease burden, and enable more effective monitoring of progress toward eliminating TB as a public health threat [8].

Molecular epidemiology plays a crucial role in the prevention and control of TB by identifying genetic diversity, determining lineage [9], tracing transmission chains [10], gathering data relevant to tracking outbreaks [10,11,12,13,14], detecting exogenous reinfection [15,16], and predicting drug resistance profiles of MTBC. Over the years, molecular techniques available for genetic analysis of MTBC isolates have included spoligotyping [17], restriction fragment length polymorphisms based on the IS*6110* element [18], mycobacterial interspersed repetitive units-variable number tandem repeat (MIRU-VNTR) typing, a widely used method based on the variability of tandem repeats within minisatellite regions of the MTBC genome that vary among isolates [10,19,20], and more recently whole genome sequencing [21,22,23,24,25], which has been routinely used in Canada since 2018.

Nearly all of the previously published molecular epidemiology studies in Canada examined MTBC isolates from large metropolitan settings in British Columbia [11,26,27,28,29,30,31], Ontario [32,33], Quebec [34,35,36], Manitoba [10,37], and Alberta [14,38], as well as smaller northern communities in Nunavut [25,39], Yukon [23] and the Northwest Territories [40]. A few studies have included regional or interprovincial isolates [41,42,43]. To date, no published study has reported comprehensive data on (1) the geographic distribution of MTBC cases in NB, (2) the demographic characteristics of TB patients in the province, (3) DST and detailed 24-loci MIRU-VNTR genotyping data, including circulating MTBC lineages in this region, or (4) the predominant anatomical site of MTBC isolation in NB. The only existing study that included genotyping data from NB assessed the performance of the 24-loci MIRU-VNTR genotyping method using isolates submitted from multiple Canadian jurisdictions over a decade [20]. Our study builds upon this work by incorporating data spanning over two decades and offers a more extensive and in-depth characterization of TBD in NB.

## 2. Materials and Methods

### 2.1. Study Setting and Design

**Figure 1:**
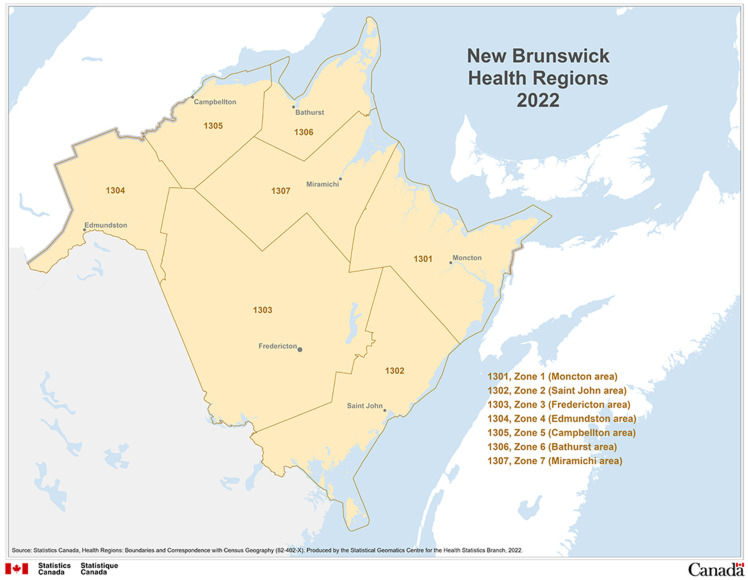
New Brunswick health regions, 2022. Source: Statistics Canada, Health Regions: Boundaries and Correspondence with Census Geography—Map 4 New Brunswick Health Regions, 2022, Catalogue no. 82-402-X (2023). 2 January 2026. Reproduced and distributed on an “as is” basis with the permission of Statistics Canada [44].

This retrospective study included all TBD patients with culture-confirmed MTBC clinical isolates in the Atlantic Canadian province of NB, from 1 January 2002 to 31 December 2024. This timeframe was chosen because it includes the period during which genotyping data for most MTBC isolates are available for NB. There are seven healthcare zones in NB, each aligned with a specific geographic region of the province (Figure 1) [44]. Cases of TB diagnosed solely on clinical grounds were excluded from the analysis.

### 2.2. Study Samples

**Figure 2:**
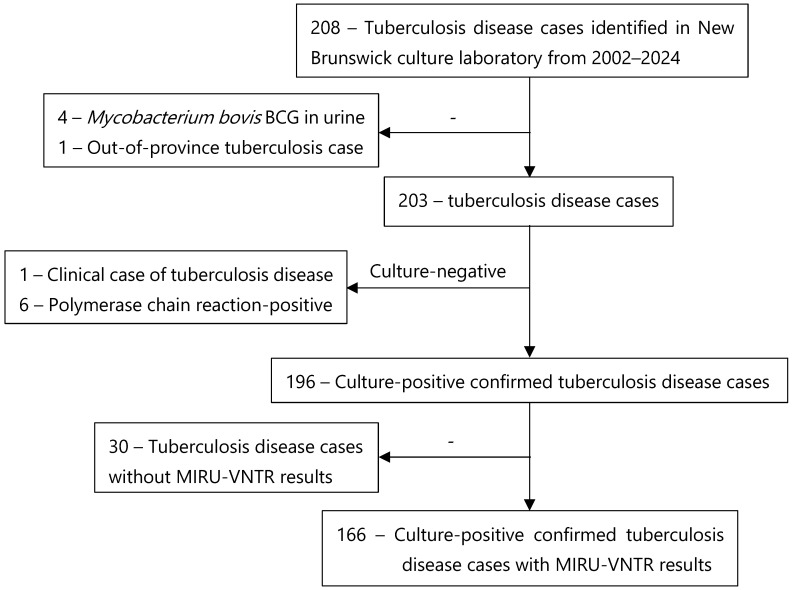
Sample enrollment and study flow chart.

Among the 208 TB cases identified during the study period, 201 were culture-positive TB cases. Five cases were excluded; one was an out-of-province case, while four involved urine specimen isolates with growth of *M. bovis* BCG, consistent with intravesical BCG therapy rather than TBD. Among the 203 TBD cases identified in the province during the study period, one was excluded because it was a clinical diagnosis, and six others were excluded because they were either culture-negative or not cultured, despite testing positive by polymerase chain reaction with a compatible clinical picture. Of the remaining 196 eligible cases, 30 were excluded because of missing MIRU-VNTR results, and the remaining 166 cases were included in the analysis (Figure 2).

Demographic and clinical laboratory data were obtained from patients’ laboratory paper records and electronic charts, including the Allscripts and Cerner Millennium databases at the Saint John Regional Hospital, which serves as the mycobacteriology reference laboratory for the province.

### 2.3. Identification, Drug Susceptibility Testing, and Genotyping

Identification, Drug Susceptibility Testing (DST), and genotyping of *M. tuberculosis* complex isolates were performed at the National Reference Center for Mycobacteriology in Manitoba, Canada, as previously described [19,21,45,46].

Briefly, MTBC isolates were identified using the BioHansel bioinformatics tool [45] while DST to first-line anti-TB drugs, rifampicin (1.0 µg/mL), isoniazid (INH) (0.1 µg/mL), pyrazinamide (PZA) (100 µg/mL), ethambutol (5.0 µg/mL), and where needed second-line anti-TB drugs, streptomycin (1.0 µg/mL), capreomycin (CAP) (2.5 µg/mL), ethionamide (5.0 µg/mL), p-aminosalicylic acid (PAS) (4.0 µg/mL), linezolid (1.0 µg/mL), moxifloxacin (0.25 µg/mL), ofloxacin (2.0 µg/mL), kanamycin (2.5 µg/mL), amikacin (1.0 µg/mL) was performed using the fluorescence proportion method on the Bactec MGIT 960 system (Becton Dickinson, Sparks, MD, USA) [19,46].

Crude DNA for 24-loci MIRU-VNTR was extracted by suspending mycobacterial cultures in 250–500 μL of Tris-EDTA buffer containing 0.5 μm silica beads, boiled for 10 min, and then sonicated in a water bath at 35 kHz for 15 min, as described previously [38]. The 24-loci MIRU-VNTR genotyping was carried out using established protocols [19,20,46,47].

Data from all patients were entered into Excel^®^ spreadsheets, de-identified, and assigned a unique number. Genetic relatedness, lineage identification, and clustering among the isolates were analyzed with the MIRU-VNTR*plus* database (http://www.miru-vntrplus.org/) using deidentified data imported from the Excel^®^ file [47,48]. MIRU-VNTR data were analyzed using the categorical coefficient and unweighted pair group method with arithmetic averages (UPGMA) and categorical distance. A cluster was defined as two or more isolates with identical 24-MIRU-VNTR patterns, while isolates with unmatched 24-MIRU-VNTR patterns were categorized as non-clustered.

The discriminatory index of each of the 24-MIRU-VNTR loci was calculated using the Hunter and Gaston Discriminatory Index (allelic diversity, *h*) [49] using a web application (http://insilico.ehu.es/mini_tools/discriminatory_power/, accessed on 24 November 2025). The HGDI ranges from 0.00 to 1.00, where a value of 0.00 signifies that all strains are identical, and a value of 1.00 indicates that each strain in the sample is different. The allelic diversity was classified as high (h > 0.6), moderate (0.3 ≤ h ≤ 0.6), or poor (h < 0.3) [50]. The clustering rate, defined as (*n*_c_ − *c*)/*n*, where *n*_c_ is the total number of clustered isolates, *c* is the number of clusters, and *n* is the total number of isolates in the sample [51], was used to determine recent transmission of MTBC [52]. It is expressed as a percentage, where lower values indicate higher discriminatory power. Descriptive statistics were used to characterise the demographic and clinical characteristics.

## 3. Results

### 3.1. Descriptive Statistics

**Figure 3:**
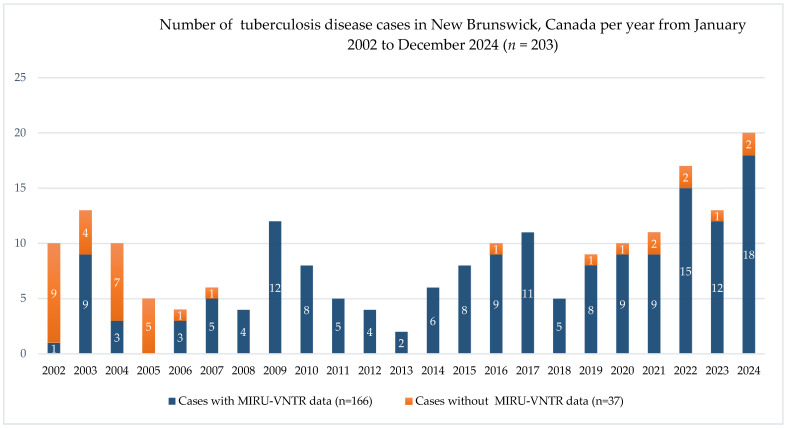
Number of tuberculosis disease cases in New Brunswick, Canada, from January 2002 to December 2024 (*n* = 203). Each bar shows the total number of cases for the corresponding year, with the colored segments within each bar indicating the number of patients with (blue) and without (orange) MIRU-VNTR data.

The temporal distribution of TBD cases identified annually in the NB laboratory from 2022 to 2024 is shown in Figure 3. The 166 MTBC isolates included for analysis in this study were obtained from 80 males and 86 females, with ages ranging from 12 to 97 years (mean age = 46.7 years). Two of the isolates were obtained from the same patient cultured seven years apart and were found to be distinct isolates, suggestive of re-infection rather than recurrence. The majority of TBD cases (*n* = 141; 84.9%) were reported from Zones 1, 2 and 3, which are the most populated regions of NB and primary settlement areas for most new immigrants.

The respiratory system was the most commonly affected anatomical site, accounting for 118 cases (71.1%). Among extrapulmonary presentations, lymphatic involvement was the most frequent, observed in 22 cases (13.1%). All the other single-site extrapulmonary cases accounted for less than 7% of the cases. Multisite extrapulmonary involvement was identified in 5 cases (3.0%). Other sample characteristics are presented in Table 1.

### 3.2. Drug Susceptibility Testing

Drug susceptibility testing results were obtained for 165 of the 166 MTBC isolates included in the study, as one isolate could not be successfully propagated for DST testing. Most of the isolates demonstrated high susceptibility to first-line anti-TB drugs, with resistance rates remaining low across the panel tested. Susceptibility was highest for ethambutol (98.1%; 159/162), rifampicin (97.6%; 161/165), and PZA (97.0%; 159/164), while INH showed slightly lower susceptibility at 90.9% (n = 150/165). Resistance to at least one first-line anti-TB agent was detected in 15 isolates, including 11 cases of mono-resistance to INH *(n* = 9) and PZA (*n* = 2). Notably, four isolates (2.4%; 4/165) were resistant to both isoniazid and rifampicin, indicative of multidrug-resistant (MDR) TB.

Among these four MDR TB isolates, a thoracic spine isolate exhibited additional resistance to PZA, ethambutol, streptomycin, ethionamide, capreomycin, PAS, kanamycin, amikacin and rifabutin; one respiratory isolate demonstrated resistance to PZA, streptomycin, ethionamide and rifabutin; another vertebral body isolate was also resistant to PZA, ethionamide and rifabutin; and a final sputum specimen showed additional resistance to ethambutol and streptomycin. All MDR TB isolates in this study were sensitive to fluoroquinolones. The MDR isolates were detected over three years. In 2015, one out of eight isolates (12.5%) was classified as MDR. In 2017, this number increased slightly, with two out of eleven isolates (18.2%) showing MDR. By 2018, the proportion rose further, with one out of five isolates (20.0%) identified as MDR. Among 37 isolates that underwent fluoroquinolone DST during the study period, only 1 isolate from a urine specimen demonstrated resistance, suggesting a low-level of fluoroquinolone-resistance in the province.

### 3.3. MIRU-VNTR Genotyping Results

Results for all 24 MIRU-VNTR loci were available for 147 of the isolates. Nineteen isolates had missing data for one (*n* = 13) or two (*n* = 6) loci. Locus 2163b had the highest number of missing alleles (*n* = 6), followed by locus 4052 (*n* = 4), 1955 (*n* = 4), and 3690 (*n* = 4). The remaining were missing either one (loci 0424, 0577, 0960, 4156, and 4348) or three loci (locus 2165) (Table 2).

The discriminatory power of the 24 MIRU-VNTR loci in this study was 0.9984. The discriminatory power of each locus ranged from 0.1046 (locus 0154) to 0.8394 (locus 4052) across all isolates. As indicated in Table 2, half of the loci (4052, 2163b, 1955, 0802, 2996, 0420, 3690, 0960, 3192, 2165, 4156, and 2401) demonstrated high discriminatory power. Seven loci (2531, 0577, 1644, 4348, 0580, 2461, and 2347) exhibited moderate discriminatory power, while five loci (2687, 2059, 3171, 3007, and 0154) had poor discriminatory power.

**Figure 4:**
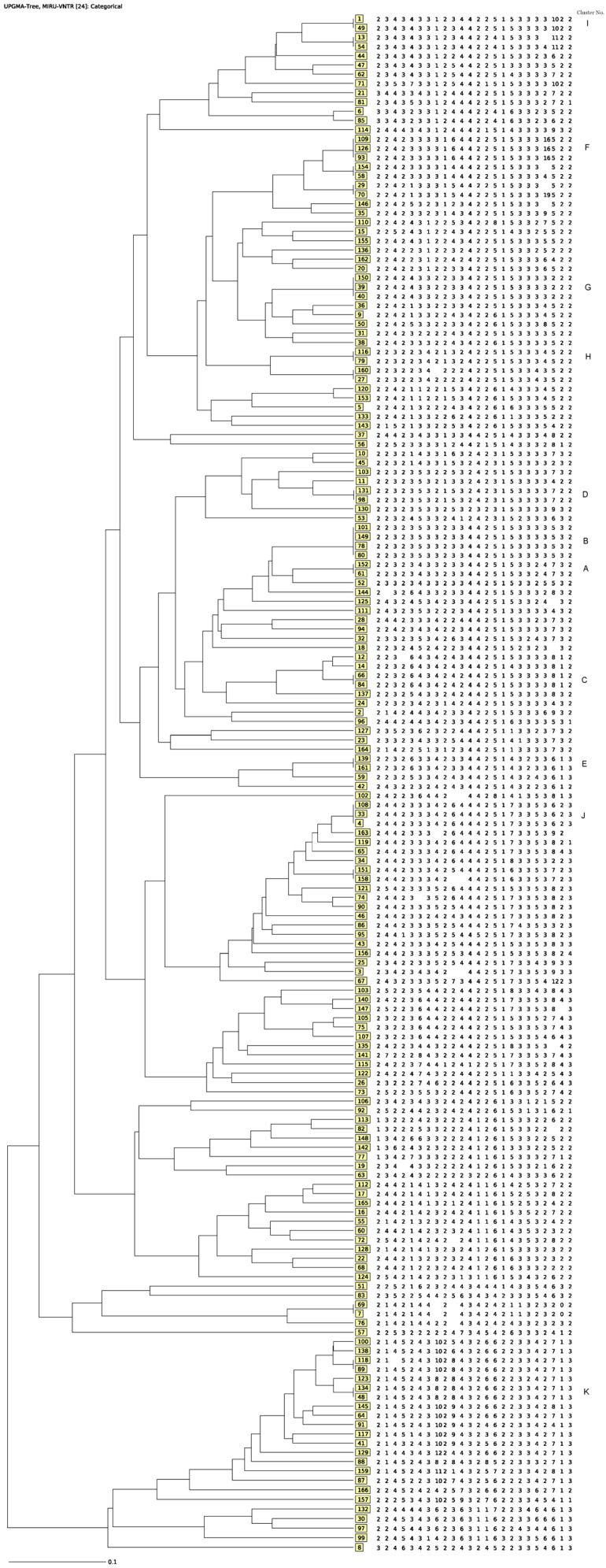
Genetic tree showing the phylogenetic relationships among 166 *Mycobacterium tuberculosis* complex clinical isolates from New Brunswick, Canada, during January 2002 to December 2024 based on 24-locus MIRU-VNTR genotyping data. The dendrogram was generated using the UPGMA clustering algorithm in the MIRU-VNTR*plus* database. The 24-digit profile for each isolate is displayed in a single row; each number indicates the number of repeats of a single locus. The 11 clusters identified in this study are identified as A–K in the figure.

Cluster analysis of the 166 MTBC isolates identified 150 distinct patterns. Among these, 27 (16.2%) isolates were grouped into 11 clusters, containing 2 isolates (7 clusters), 3 isolates (3 clusters), and 4 isolates (1 cluster), resulting in a clustering rate of 20.4%. The remaining 139 isolates had unique genotypes. Among these, seven pairs of isolates were not considered clustered because one or both isolates in each pair lacked one or two loci, despite most loci matching (Figure 4). Likewise, three other closely related pairs of isolates had 24-MIRU-VNTR patterns that differed at only a single locus and were therefore not classified as clustered.

Among the 166 isolates analyzed, 160 were classified as *M. tuberculosis* sensu stricto, five as *M. africanum*, and one as *M. bovis*. The *M. bovis* was isolated from the gastrointestinal tract of a patient with a clinical picture compatible with TBD. This patient grew up on a cattle farm in Latin America and reported consuming unpasteurized milk while living on that farm years prior. The most common genetic lineage of *M. tuberculosis* identified was the Cameroon type with 27 isolates (16.4%). This was followed by the East African Indian (EAI) and Haarlem lineages, each with 23 isolates (13.9%), and the Beijing type with 22 isolates (13.3%). Other identified lineages included Latin American-Mediterranean (LAM) with 20 isolates (12.1%), S with 13 isolates (7.9%), Delhi/Central Asian Strain (CAS) with 12 isolates (7.3%), and X with 8 isolates (4.9%). Less frequently observed lineages were H37Rv with 5 isolates (3.0%), Ghana with 4 isolates (2.4%), West African 1 with 3 isolates (1.8%), and West African 2 with 2 isolates (1.2%). Additionally, single isolates (0.6% each) of Uganda II, Ural, and Turkey (TUR) lineages were also detected. Of the four MDR isolates identified in this study, two were of the Beijing genotype, one belonged to the LAM lineage, and the fourth to the Ghana lineage.

Of the 11 clusters identified in this study, four clusters included at least two isolates from different patients cultured within a week (Cluster G) or 3 weeks (Clusters B, C, and J). One cluster included two isolates cultured within 5 months (Cluster E). The isolates in the remaining clusters were cultured at least one year apart. Isolates in Clusters E, F, and J were cultured from patients residing in the same respective healthcare zones. All the other clusters contained at least one isolate from patients residing in different health zones. All clustered isolates were pan-susceptible to all drugs tested. No additional data were available to help establish transmission chains among these TB patients (Table 3, Figure 4). Among the non-clustered isolates, two were cultured from the same patient in 2017 and 2024. These two isolates exhibited distinct 24-loci MIRU-VNTR profiles and belonged to the Haarlem and Cameroon lineages, respectively, suggesting a second infection rather than reactivation of the previous infection.

## 4. Discussion

This study is the first to provide an in-depth overview of TBD in NB spanning over two decades. The distribution of TBD cases in NB reflects the population sizes of each geographical zone and is aligned with areas where most immigrants from regions of high TB-endemicity, who account for the majority of TBD cases in NB, tend to settle. Our study identified a likely case of TBD reinfection occurring seven years after the initial episode, based on 24-loci MIRU-VNTR genotyping. This represents the first documented case of TBD reinfection in NB and highlights the critical role of molecular epidemiology in understanding TB transmission dynamics. Throughout the study period, TB drug resistance rates in the province generally remained below the national average, except for MDR rates in 2017 (25.0%) and 2018 (12.5%). The elevated MDR rates, though based on small sample sizes, contrast with the national MDR TB rate in Canada, which peaked at only 1.8% between 2018 and 2023 [2,53]. The overall low anti-TB drug resistance in NB may be an indicator of a good surveillance program in the province. This is particularly encouraging given that drug-resistant TBD is more challenging and expensive to treat than drug-susceptible TBD [1].

The clustering rate reported herein is considerably lower than the 62% reported in an earlier Canadian study that analyzed samples from British Columbia, Saskatchewan, Manitoba, and Quebec [43]. This difference could be attributed to higher transmission patterns in larger and more densely populated cosmopolitan areas compared to the more sparsely populated NB. Given that TBD in Canada in more recent years has been found to mostly affect immigrant populations, the relative predominance of specific *M. tuberculosis* lineages in NB (i.e., EAI, Beijing, LAM, and Haarlem), compared to other regions of the country [20], may indicate that the majority of immigrants to NB originate from a limited set of geographic regions where these lineages are commonly found. The relatively low clustering rate observed in this study indicates that the majority of the TBD cases in NB are not the result of recent transmission events. However, considering the majority of TBD cases in this study were of a respiratory nature and TB is almost exclusively transmitted through the airborne route, there remains a risk of local transmission. As such, the MIRU-VNTR genotyping can assist public health with tracking and may guide interventions in certain settings.

Consistent with previous reports in Canada that assessed the effectiveness of the 24-MIRU-VNTR locus method for genotyping MTBC isolates [20,46], the current study found that this method demonstrated a high discriminatory power in distinguishing isolates from NB. The recommendation to use different sets of MIRU-VNTR loci for improved differentiation of isolates from specific MTBC lineages, such as Beijing and EAI, was not needed in our context because the 24-MIRU-VNTR loci performed well across all lineages identified in our study, and no single lineage was overrepresented in our sample [54]. However, in some regions of Canada, such as Nunavut, alternative genotyping methods with higher discrimination, like whole genome sequencing, may be necessary to understand MTBC transmission chains better, as the 24-MIRU-VNTR loci panel may be insufficient due to the predominance of highly homogeneous MTBC genotypes and may not provide adequate discrimination in clonal outbreaks [11].

The gradual but sustained rise in TBD cases in NB over recent years parallels an increase in the foreign-born population in NB. While the overall TB incidence in NB remains lower than the national average, the proportion of TBD cases among individuals born outside Canada increased substantially over the past two decades, reflecting growing immigrant settlement in the region [4,7]. This pattern mirrors national trends, where foreign-born individuals account for most of the TBD diagnoses and face a higher risk due to TB infection (TBI) acquired before migration, often reactivating after arrival [2,6,55].

Fewer than 3% of TBD cases among immigrants in Canada are identified through pre-arrival or post-landing screening, with the majority stemming from reactivation of undiagnosed TBI (55). These trends suggest that the changing demographics in NB, where a rising proportion of residents come from regions of higher TB endemicity, may be contributing to the observed increase in TBD cases. Improving TBI screening and preventive interventions within settlement and primary care programs could help mitigate these demographic influences and support TB elimination efforts in low-incidence settings such as NB.

### Limitations

This study has certain limitations. First, the lack of patient-level clinical data, such as comorbidities and treatment outcomes, restricted our analyses to laboratory information obtained from the provincial reference laboratory. Clinical data are not routinely linked to laboratory submissions by requesting healthcare providers and are not systematically accessible across institutions or extended time periods, limiting the ability to assess clinical characteristics or outcomes. Second, the incomplete availability of MTBC isolates and DST results for all cases during the study period hindered a comprehensive assessment of TBD in NB. While this could potentially introduce selection bias, the relatively few exclusions are unlikely to have a significant effect on the interpretation of the present results. Finally, the lack of classical epidemiological data, such as contact-tracing or information about patient movement, hindered confirmation of transmission links among the clustered isolates and a more detailed description of possible transmission dynamics. Future studies should address this shortcoming.

## 5. Conclusions

In conclusion, this study has provided initial insights into the landscape of TBD in NB over the past two decades, including identifying a probable first TBD reinfection case in NB. The findings indicate that the incidence of TBD in the province has consistently been below the national average, with drug resistance and transmission levels generally remaining low. Our findings provide valuable preliminary data that can serve as a foundation for guiding future research on the epidemiology of TBD in the province.

## Figures and Tables

**Table 1:** Characteristics of tuberculosis cases in New Brunswick, Canada, with genotyping results from 2002 to 2024 (*n* = 166).

Characteristics	Biological Sex		Total (Percentage)
	M	F	
Age	80	86	166 (100)
Age Group			
<15	0	2	2 (1.2)
15–24	17	17	34 (20.5)
25–34	17	17	34 (20.5)
35–44	3	12	15 (9.0)
45–54	14	9	23 (13.9)
55–64	7	7	14 (8.4)
>65	22	22	44 (26.5)
Number of isolates per healthcare zone			
Zone 1 (Moncton area)	40	37	77 (46.4)
Zone 2 (Saint John area)	10	20	30 (18.1)
Zone 3 (Fredericton area)	14	20	34 (30.5)
Zone 4 (Edmunston area)	3	3	6 (3.6)
Zone 5 (Campbellton area)	5	3	8 (4.8)
Zone 6 (Bathurst area)	6	2	8 (4.8)
Zone 7 (Miramichi)	2	1	3 (1.8)
Anatomical isolation site:			
Respiratory	55	63	118 (71.1)
Lymphatic	9	13	22 (13.3)
Musculoskeletal	9	2	11 (6.6)
Genitourinary	2	2	4 (2.4)
Gastrointestinal	2	2	4 (2.4)
Central nervous system	0	1	1 (0.6)
Respiratory, Lymphatic	1	0	1 (0.6)
Respiratory, Gastrointestinal	1	0	1 (0.6)
Respiratory, Musculoskeletal	0	1	1 (0.6)
Respiratory, Musculoskeletal, Genitourinary	0	1	1 (0.6)
Respiratory, Genitourinary, Central nervous system	1	0	1 (0.6)

**Table 2:** Allele diversity of *Mycobacterium tuberculosis* complex isolates from New Brunswick, Canada, from January 2002 to December 2024.

Locus	Number of Isolates with Identified MIRU-VNTR Alleles																Allelic Diversity	Designation
	0	1	2	3	4	5	6	7	8	9	10	11	12	16	19	NA		
4052			11	4	10	37	24	38	26	6	3	2	1			4	0.8394	High
2163b		2	31	36	35	26	16	2	6	5						7	0.8279	High
1955		13	31	50	38	9	4		4		11	1	1			4	0.8026	High
802		27	33	65	26	5	8	2									0.7574	High
424		23	68	30	36	7		1								1	0.7327	High
2996		6	23	5	20	80	11	18	3								0.7197	High
960		2	5	71	55	19	9	3	1							1	0.6902	High
3690		3	43	77	19	8	5	1	1	1				3	1	4	0.6899	High
3192			24	81	24	34	3										0.6819	High
2165		2	30	51	72		5	2		1						3	0.676	High
4156	2	35	83	33	12											1	0.6605	High
2401		21	81	1	62	1											0.61	High
2531				7	10	98	47	2	2								0.569	Moderate
577			17	36	103	8	1									1	0.5531	Moderate
1644		12	26	107	20	1											0.5435	Moderate
4348		5	107	52	1											1	0.4821	Moderate
580		1	126	16	3	19	1										0.4035	Moderate
2461		16	128	2	3	4	11	1	1								0.393	Moderate
2347		1	8	31	126												0.389	Moderate
2687		138	27	1													0.2841	Poor
2059		23	143														0.2402	Poor
3171		2	6	149	2	7											0.1921	Poor
3007		4	6	154	2												0.1382	Poor
154		5	157	4													0.1046	Poor

NA: No identifiable alleles.

**Table 3:** Description of participants with clustered *Mycobacterium tuberculosis* isolates from New Brunswick, Canada, from January 2002 to December 2024 (*n* = 27).

Cluster	Patient ID	Isolation Year	Age	Sex	Anatomical Site	Region of Isolation
A	61	2003	66	F	Respiratory	3
	152	2016	67	F	Respiratory	6
B	78	2017	25	F	Respiratory	1
	80	2017	65	F	Respiratory	1
	149	2019	64	M	Respiratory	1
	101	2023	27	F	Respiratory	2
C	84	2016	74	M	Respiratory	2
	66	2016	76	M	Respiratory	7
D	98	2022	24	F	Lymphatic	4
	131	2024	46	F	Respiratory	1
E	161	2020	26	F	Respiratory	5
	139	2021	50	F	Respiratory	5
F	93	2021	36	F	Lymphatic	1
	109	2022	25	M	Musculoskeletal	1
	126	2023	19	F	Respiratory	1
G	39	2010	88	M	Respiratory	3
	40	2010	50	M	Respiratory	1
	150	2020	69	M	Respiratory	1
H	116	2022	26	F	Respiratory	1
	79	2024	30	F	Respiratory	1
I	1	2004	77	F	Respiratory	4
	49	2013	85	F	Musculoskeletal	1
J	108	2009	70	F	Respiratory	2
	4	2009	61	F	Respiratory	2
	33	2010	63	F	Respiratory	1
K	48	2022	51	M	Respiratory	2
	134	2024	56	M	Respiratory	1

## Data Availability

The data presented in this study are openly available in Open Science Framework at https://doi.org/10.17605/OSF.IO/MS37W (accessed on 2 January 2026).

## Abbreviations

The following abbreviations are used in this manuscript:

BCG	Bacillus Calmette–Guérin
CAP	Capreomycin
CAS	Central Asian Strain
DNA	Deoxyribonucleic Acid
DST	Drug Susceptibility Testing
EAI	East African Indian
HGDI	Hunter and Gaston Discriminatory Index
INH	Isoniazid
LAM	Latin American-Mediterranean
MDR	Multidrug-resistant
MGIT	Mycobacteria Growth Indicator Tube
MIRU-VNTR	Mycobacterial Interspersed Repetitive Unit-Variable Number Tandem Repeat
MTBC	*Mycobacterium tuberculosis* complex
NB	New Brunswick
PAS	p-aminosalicylic acid
PZA	Pyrazinamide
TBD	Tuberculosis disease
TBI	Tuberculosis infection
TUR	Turkey
UPGMA	Unweighted Pair Group Method with Arithmetic Averages
WHO	World Health Organization
